# Antitumorigenic effect of atmospheric-pressure dielectric barrier discharge on human colorectal cancer cells via regulation of Sp1 transcription factor

**DOI:** 10.1038/srep43081

**Published:** 2017-02-22

**Authors:** Duksun Han, Jin Hyoung Cho, Ra Ham Lee, Woong Bang, Kyungho Park, Minseok S. Kim, Jung-Hyun Shim, Jung-Il Chae, Se Youn Moon

**Affiliations:** 1 Department of Applied Plasma Engineering, Chonbuk National University, 567 Baekje-daero, Jeonju, Jeollabuk-do, Republic of Korea; 2 Department of Dental Pharmacology, School of Dentistry and Institute of Oral Bioscience, BK 21 Plus, Chonbuk National University, 567 Baekje-daero, Jeonju, Jeollabuk-do, Republic of Korea; 3 Department of New Biology, DGIST, Daegu 42988, Republic of Korea; 4 Department of Pharmacy, College of Pharmacy and Natural Medicine Research Institute, Mokpo National University, 1666 Yeongsan-ro, Muan-gun, Jeonnam, Republic of Korea; 5 Department of Quantum System Engineering, Chonbuk National University, 567 Baekje-daero, Jeonju, Jeollabuk-do, Republic of Korea

## Abstract

Human colorectal cancer cell lines (HT29 and HCT116) were exposed to dielectric barrier discharge (DBD) plasma at atmospheric pressure to investigate the anticancer capacity of the plasma. The dose- and time-dependent effects of DBDP on cell viability, regulation of transcription factor Sp1, cell-cycle analysis, and colony formation were investigated by means of MTS assay, DAPI staining, propidium iodide staining, annexin V–FITC staining, Western blot analysis, RT-PCR analysis, fluorescence microscopy, and anchorage-independent cell transformation assay. By increasing the duration of plasma dose times, significant reductions in the levels of both Sp1 protein and Sp1 mRNA were observed in both cell lines. Also, expression of negative regulators related to the cell cycle (such as p53, p21, and p27) was increased and of the positive regulator cyclin D1 was decreased, indicating that the plasma treatment led to apoptosis and cell-cycle arrest. In addition, the sizes and quantities of colony formation were significantly suppressed even though two cancer promoters, such as TPA and epidermal growth factor, accompanied the plasma treatment. Thus, plasma treatment inhibited cell viability and colony formation by suppressing Sp1, which induced apoptosis and cell-cycle arrest in these two human colorectal cancer cell lines.

Cold atmospheric-pressure plasmas (CAPs) have been intensively studied for a variety of biological and clinical applications, including wound healing, tissue sterilization, blood coagulation, tooth bleaching, and antitumor properties^1^,^2^,^3^,^4^,^5^. In general, CAPs show the characteristics of low gas temperatures, similar to those of room temperatures, which are advantageous in preventing harmful thermal damage to cells or tissues during plasma treatment. Many research groups have studied the mechanism of interaction between CAPs and biological materials based on the pioneering work of Eva Stoffels, whose description of the “plasma needle” first revealed the potential of CAPs as an alternative therapeutic tool in the field of biomedicine^6^. Although plasma chemistry is complex and its physical influence on biological cells remains to be clarified, both the reactive oxygen species (ROS) (e.g., O, OH, O_2_^−^, H_2_O_2_, and O_3_) and the reactive nitrogen species (RNS) (e.g., NO, NO_2_, HNO_2_, and ONOOH) that are produced in CAPs are believed to be important factors in biomedical applications^7^,^8^,^9^,^10^. Moreover, charged particles (e.g., electrons and ions) and ultraviolet (UV) radiation are also generated in CAPs and can affect living cells. These physical and chemical properties of plasma are now being actively studied to evaluate their potential anticancer effects^11^,^12^,^13^.

Conventionally, anticancer drugs that induce apoptosis have been developed as an outgrowth of chemotherapy^14^,^15^,^16^. For example, the antitumor effect of honokiol was reported for human oral squamous cancer cell lines HN22 and HSC4^16^. Several drugs that produce ROS in cancer cells also result in cell-cycle arrest or apoptosis^17^,^18^. With this in mind, many research groups have used plasma treatment to determine its effects on various types of cancer cells by inducing concentrations of ROS sufficient to cause cell-cycle arrest and apoptosis^19^,^20^,^21^,^22^,^23^,^24^,^25^,^26^. Changing the duration of the dose or the reactive radical density by adjusting the rate of gas flow, the applied power, and the design of the source has been used to estimate the critical oxidative stress level of cancer cells. In particular, the addition of oxygen gas was successful in many studies because it allowed the level of ROS induced by the plasma treatment to be increased in a controlled manner^27^,^28^. Also, both intracellular and extracellular ROS levels have been examined relative to cell proliferation and any damage to lipids, proteins, and DNA^29^,^30^. Thus, CAPs would appear to be a suitable alternative tool for achieving these effects in cancer cells.

Most of the studies alluded to above used jet-type atmospheric-pressure plasma sources to treat the cancer cells. Jet-type CAPs are preferable for treatments that involve direct contact with biological structures, such as for skin regeneration or wound healing. However, cancer cells are normally contained in a liquid culture medium for the purpose of *in vitro* diagnostic testing. Typically, in the biological research setting, a standard-size Petri dish is used to contain and cultivate these cells. Thus, we mounted a dielectric barrier discharge apparatus to a Petri dish that was 100 mm in diameter to uniformly treat whole cancer cells. ROS and RNS are produced within the discharge area and are melted in the medium, thus reaching biomolecules^31^. Compared with the needle-like jet-type plasma delivery system, our Petri dish sized DBD (PDBD) as seen in Fig. 1 was more appropriate for treating a large area at once. Thus, the need to collect cells in a specific area, including plasma-treated cells, can be avoided.

Colorectal cancer (CRC) was first reported as a type of ulcerative colitis, and today it is being diagnosed in more than 1.2 million people a year worldwide^32^. This common, high-risk disease results in 600,000 deaths annually^33^. As is true for other types of cancer, a wide variety of compounds are being studied in an attempt to reduce cancer cell growth and to find a mechanism that will induce apoptosis. For example, atorvastatin, γ-tocotrienol, and celecoxib are reported to have a synergistic effect on human CRC cells in which they inhibit cancer cell growth by inducing G_0_/G_1_-phase cell-cycle arrest and apoptosis^34^. The human microRNA-21 is one of the CRC cell activators that enhance the cells’ capacity to invade, and programmed cell death 4 (pdcd4) proteins (also known as neoplastic transformation inhibitor) is reported to inhibit activation of CRC cells by microRNA-21^35^. Interestingly, another report states that ascorbic acid leads to ROS-dependent repression of transcription factor Sp1 and Sp-regulated genes in colon cancer cells^36^. Transcription factor Sp1 playing an important role as basal transcription factor is a protein involved in cell-cycle progression and apoptotic cell death^14^,^15^. Therefore, these studies suggest that plasma, being rich in ROS, may represent an important gateway to treating cancer.

Therefore, we investigated the effect of PDBD on transcription factor Sp1 in the human CRC cell lines HT29 and HCT116 to evaluate the anticancer capacity of such treatment depending on dose times. In addition, we assessed the electrical characteristics of PDBD to determine the optimal conditions for treating CRC cells, as well as the rotational temperature, considering that the gas temperature at atmospheric pressure can be measured by means of OH molecular emission spectra analysis. Cell viability and morphological aspects were also examined to evaluate the effects of growth inhibition in relation to dose time. Cells that stained positive for annexin V–FITC were quantitated, and several important proteins related to cell growth and cell cycles were analyzed using Western blotting and reverse transcription polymerase chain reaction (RT-PCR). Lastly, the antitumor effect of PDBD was supported by the changes in colony size when two tumor promoters, tetradecanoyl phorbol acetate (TPA) and epidermal growth factor (EGF), were injected into the CRC cells.

## Results

### Electrical characteristics of plasma

Figure 2A presents a typical waveform of the applied voltage and current when a root-mean-square voltage (V_rms_) of 1 kV was applied at the electrode. The measured peak-to-peak voltage (V_pp_) and current (I_pp_) were 4.7 kV and 100 mA, respectively. The duty ratio of pulse-like voltage was 50%, and the pulse width was 5 μsec. During a half-cycle of voltage, two peaks of current appeared in both positive and negative voltage, as shown in Fig. 2C,D, respectively. In Fig. 2C, the first peak of current increased to 50 mA simultaneously with an increase in the applied voltage. A sufficiently intense electric field from the electrode led to this first current peak as a result of gas breakdown and ionization. Plenty of electrons then accumulated on the surface of the dielectric quartz, which increased the potential difference between the electrode and the dielectric surface. Eventually, a new discharge was propagated on the dielectric quartz, evident as the second current peak (Fig. 2C). Two currents peaks of opposite polarity were also observed in the negative phase of the applied voltage, as shown in Fig. 2D. This phenomenon is similar to that seen in other studies using pulse-power dielectric barrier discharge^37^,^38^. A slight difference in this study was use of a waveform that is not a typical conventional pulse, which is flat initially but falls immediately after a period of rising period, meaning that the “silent period” of discharge is absent. The consumed power was a product of voltage and current divided by one period, as plotted in Fig. 2B. The power was almost linearly increased from 0.6 to 7.0 W as the V_rms_ was adjusted between 370 and 1,000 V. In this case, the glow discharge spread to the center, without streamers (Fig. 3A–C). When V_rms_ was greater than 1 kV, high-current streamers appeared, and the consumed power began to increase rapidly (see Fig. 2B). PDBD uniformly covered the dielectric quartz with 1 kV, which is a favorable condition for treating CRC in the Petri dish with a single exposure. Figure 3D shows an image of discharge when the PDBD was not mounted to the Petri dish. In this case, the glow region was limited to the edge of the electrodes at any value of V_rms_.

### Optical emission spectroscopy of plasma

Figure 4A presents optical emission spectra in wavelengths ranging from 280 to 850 nm, in which the atom lines of helium (He) I (501.5, 587.5, 667.8, and 706.5 nm), oxygen (O) I (777.2 nm), and hydrogen (H) I (656.1 nm) are dominant. H_β_ of 486.1 nm was weakly observed. In a collisional radiative process, metastable He (2^3^S) plays an important role through Penning ionization. For example, the He atom line is dominant from a collisional radiative process owing to electron-impact excitation of metastable He rather than high-energy electron impact ionization of ground-state He^39^. Large numbers of low-energy electrons can participate in this process because the threshold energy needed to excite metastable He is 2.9 eV. In atmospheric-pressure conditions, high-energy electrons comparable to the threshold energy (22.7 eV) for single-step He ionization are barely present owing to a short mean free path. Possible sources of the O atom lines are the dissociation of oxygen gas and water vapor in ambient air. Metastable He also plays an important role in this process, as follows:


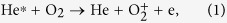



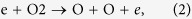



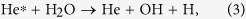



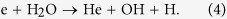


Molecular spectra such as OH (A^2^∑^+^ − X^2^Π), N_2_ second positive system (SPS, C^3^Π_u_–B^3^Π_g_), and N_2_^+^ first negative system (FNS, 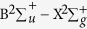) are shown in Fig. 4B. Several vibrational band heads of N_2_ SPS spectra can be observed at 337.1, 353.6, 357.6, 375.5, and 380.4 nm, which were induced by ambient N_2_ molecules. N_2_ FNS molecular lines are driven by the Penning ionization by metastable He. To estimate rotational temperature, OH molecular emission lines were obtained under the same conditions as those used for treating CRC, which are analyzed using a synthetic spectrum method^40^. The rotational temperature is close to a gas temperature in the atmospheric-pressure plasma. When PDBD is sustained with 1 kV, the rotational temperature is 340 K (Fig. 3C). The injection of He gas through two holes on the top side, along with the two venting paths, allowed efficient, uniform gas flow inside the discharge area. Thus, under these conditions, considerable thermal damage does not result from PDBD.

### PDBD inhibits cell viability and increases apoptosis in CRC cells

Viability of the HT29 and HCT116 cells was investigated with each change in plasma dose times (15, 30, 60, 120, and 180 sec). An MTS assay was used to compare cell viabilities between those conditions, including non-treated (control) CRC cells, at two time points—24 and 48 h—as shown in Fig. 5A,B. In both cell lines, viability decreased as dose time increased. Although the reduction did not change significantly between the two time points (24 h and 48 h), with the shorter dose times (i.e., 15 and 30 sec) the difference became more significant as time passed (i.e., when the dose time exceeded 60 sec). These results were verified on images of morphological changes (Fig. 5C). Since DAPI specifically stains nuclei, this method was used to confirm the induction of apoptosis by PDBD in the HT29 and HCT116 cells. The results showed fragmented and condensed nuclei in the cells treated with plasma (for 30, 60, and 120 sec) and then incubated for 48 h, as compared with the control nuclei. Thus, PDBD inhibited the growth of CRC cells.

In addition, we used propidium iodide and annexin V staining to examine the apoptotic effect on the HT29 and HCT116 cells after 48 h had elapsed (Fig. 5D,E, respectively). In Fig. 5D, the proportions of early apoptotic cells in the HT29 group were 2.52 ± 0.67%, 7.35 ± 0.93%, 16.70 ± 0.48%, and 45.73 ± 0.97% when dose times were 0, 30, 60, and 120 sec, respectively, and the proportions of dead and late apoptotic cells were 0.01 ± 0.93%, 2.18 ± 1.18%, 9.36 ± 1.63%, and 30.75 ± 2.01% when dose times were 0, 30, 60, and 120 sec, respectively. The proportion of live cells in the control group was 97.42 ± 0.06%, and the proportions of treated cells were reduced to 90.28 ± 0.07%, 71.34 ± 0.04%, and 21.3 ± 0.08% when the dose times were 30, 60, and 120 sec, respectively. Figure 5E shows the corresponding proportions for the HCT116 group. Those for the early apoptotic cells were 0.82 ± 2.87%, 3.19 ± 1.22%, 48.9 ± 2.24%, and 41.2 ± 1.79% when dose times were 0, 30, 60, and 120 sec, respectively; those for the dead and late apoptotic cells were 0.14 ± 1.39%, 10.82 ± 1.38%, 17.26 ± 2.03%, and 37.59 ± 1.81% when dose times were 0, 30, 60, and 120 sec, respectively. The proportion of live cells in the control were 99.00 ± 0.08, and the proportions of the treated cells were reduced to 84.98 ± 0.10%, 33.78 ± 0.06%, and 21.00 ± 0.04% when dose times were 30, 60, and 120 s, respectively. The total populations of apoptotic cells, including dead, early, and late apoptosis, increased along with increasing dose times.

### PDBD-regulated Sp1 protein levels in CRC cells

Transcription factor Sp1 is a protein involved in cell-cycle progression and apoptotic cell death^11^,^12^. The evolution of Sp1 levels according to dose times of PDBD was evident on western blot analysis (Fig. 6A). In both the HT29 and HCT116 cell lines, Sp1 protein levels (in relation to β-actin levels) were reduced with increasing dose times. Constant β-actin levels ensure that the same quantity of proteins is loaded in the analysis, and the Sp1 level was significantly decreased when the dose time was 120 sec. We also used reverse transcription polymerase chain reaction (RT-PCR) analysis to ensure the expression level of Sp1 related to mRNA (Fig. 5B). Decreasing the Sp1 protein level with increasing dose time coincided with both western blot and RT-PCR analysis. Furthermore, various apoptosis-related proteins, such as caspase 3, PARP, and the cleaved forms of each, were investigated, including Sp1 when the dose time was 120 sec (Fig. 6C). In both cell lines, a decrease in caspase 3 and an increase in cleaved caspase 3 were observed over time, indicating apoptotic progression. Cleaved PARP is a well-known marker of apoptosis, and it appeared faintly at 48 h in both cell lines. The level of Sp1 was minimal after the 120-sec dose time, relative to the levels at shorter dose times (Fig. 6A). Consistent with these observations, the immunocytochemical results revealed decreased Sp1 levels in a dose time–dependent manner in both the HT29 and the HCT116 cell lines (Fig. 6D). We conclude that suppression of Sp1 by PDBD treatment leads to apoptotic cell death.

### PDBD-regulated expressions of cell-cycle arrest and migration in CRC cells

Cell-cycle analysis was conducted to investigate the effects of PDBD on the HT29 and HCT116 cell lines. Phases such as G1/G0, S, and G2/M represent the different steps during cell proliferation. We analyzed the DNA content of the control cells and the PDBD-treated cells (for 30, 60, and 120 sec) during the 24 h after PDBD treatment to determine the minimum time that corresponded to sufficient cell replication (Fig. 7A,B). In both cell lines, the two typical peaks of G1/G0 and S were observed for the control cells (Fig. 7A). With increasing dose times, the sub-G1 phase began to appear, indicating debris due to the apoptosis. The proportion of sub-G1 gradually increased as the total proportion in cell cycling (such as G1/G0, S, and G2/M) was reduced as the dose time increased from 30 to 120 sec. Also, a proportion of G1/G0 increased to about 80% with 30 sec of PDBD treatment, as compared with the control cells. This means that PDBD treatment induces cell-cycle arrest because a substantial proportion of the G1/G0 phase could not progress to the following S phase. Next, we examined the proteins p53, p21, p27, and cyclin D1, which are involved in advancing the cell cycle from G1/G0 to the next phase (Fig. 7C). The negative cell-cycle regulation proteins, such as p53, p21, and p27, increased in both cell lines from 0 to 48 h after 120 sec of PDBD treatment. On the other hand, cyclin D1, the positive cell-cycle regulation protein, decreased as time elapsed. These changes in the levels of cell-cycle regulation proteins over time indicated cell-cycle arrest and apoptotic cell death as a result of the plasma treatment.

Figure 7D reveals a degree of cell migration with the 30-sec PDBD treatment, which is similar to that seen with *in vitro* metastasis. Both control cell lines had almost recovered at 72 hours after scratching the center of the monolayer (Fig. 7D). On the other hand, for plasma treated cases, the ratio of recovered area to the initial area slightly increased to 10% at 24 h and reached about 40% at 72 h (Fig. 7E). Based on these results, we strongly believe that the PDBD-treated HT29 and HCT116 cells were possibly within the range of proliferation, whereas the expression of the various proteins tested above was changed.

### PDBD significantly suppressed TPA- or EGF-induced CRC cell transformation

We examined the inhibitory activities of PDBD on TPA- or EGF-induced HT29 and HCT116 cells transformation. Treatment with plasma (for 30, 60, and 120 sec) significantly inhibited 50 to 90% of neoplastic transformation, as compared with TPA- or EGF-induced transformation without plasma. These results indicated that PDBD was a potent inhibitor of TPA- or EGF-induced transformation in the HT29 and HCT116 cells (Fig. 8A,C). The increased colony formation was definitely inhibited with increasing plasma dose times, and this inhibition was seen more clearly when one compares the colony size of the controls (untreated) and the 120-sec treated cells and those exposed to TPA (Fig. 8B,D). In the case of EGF, the results were similar to those seen with TPA exposure that is, colony formation was suppressed and colony size was also reduced.

## Discussion

**Figure 1:**
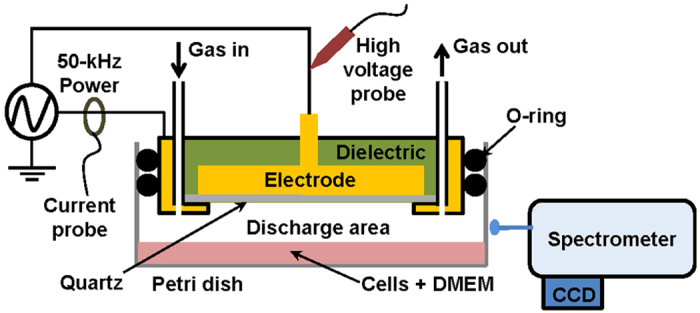
Schematic diagram of atmospheric-pressure dielectric barrier discharge source and diagnostic system, including high-voltage probe, current probe, and optical emission spectroscopy. A 50-kHz pulse-like power is applied to the disc-shaped copper electrode. The 100-mm-diameter Petri dish containing the cells and culture medium can be replaced to allow experimental conditions to be changed. (*See color version online.*).

**Figure 2:**
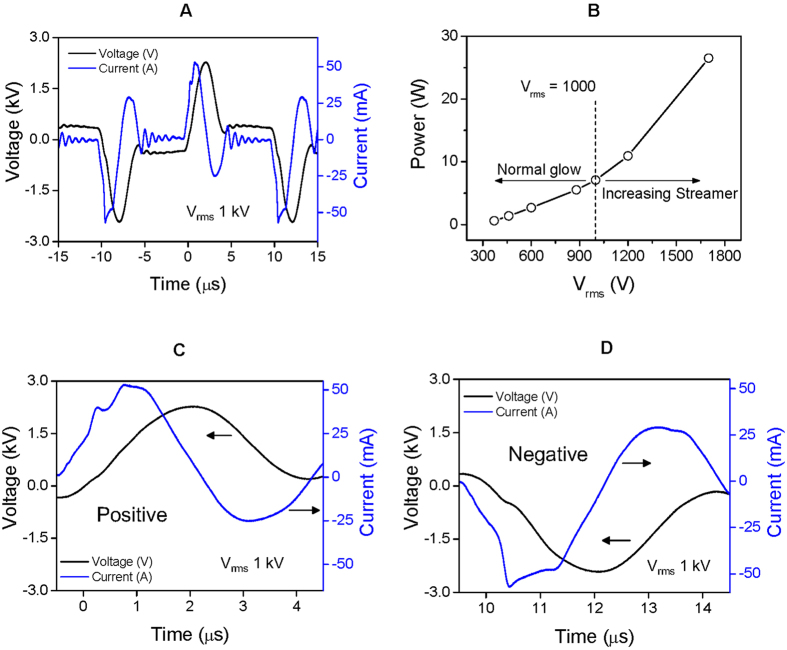
(**A**) Typical waveform of voltage (black line) applied to the electrode and current (blue line) when V_rms_ is 1 kV. (**B**) Consumed power to the applied voltage. High-current streamers begin to appear when the voltage exceeds 1 kV. During a single polarity of both positive (**C**) and negative (**D**) periods, two peaks of current can be seen. (*See color version online.*).

**Figure 3:**
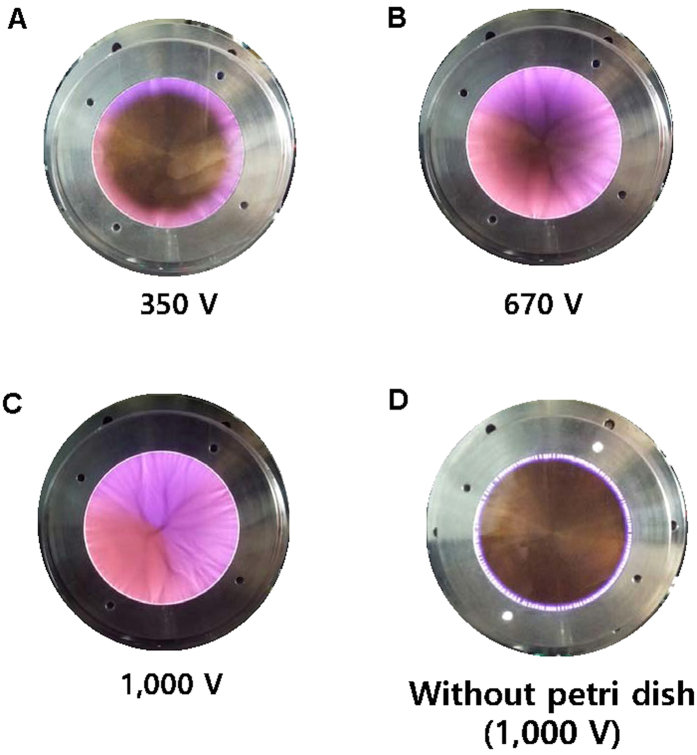
(**A**) A glow discharge can be observed at the edge of the electrode when the voltage is 350 V. The area of glow discharge is extended (**B**) and finally covers the whole electrode as voltage increases (**C**). (**D**) Without the Petri dish, the glow discharge is not able to spread into the center of the electrode at all voltage ranges. (*See color version online.*).

**Figure 4:**
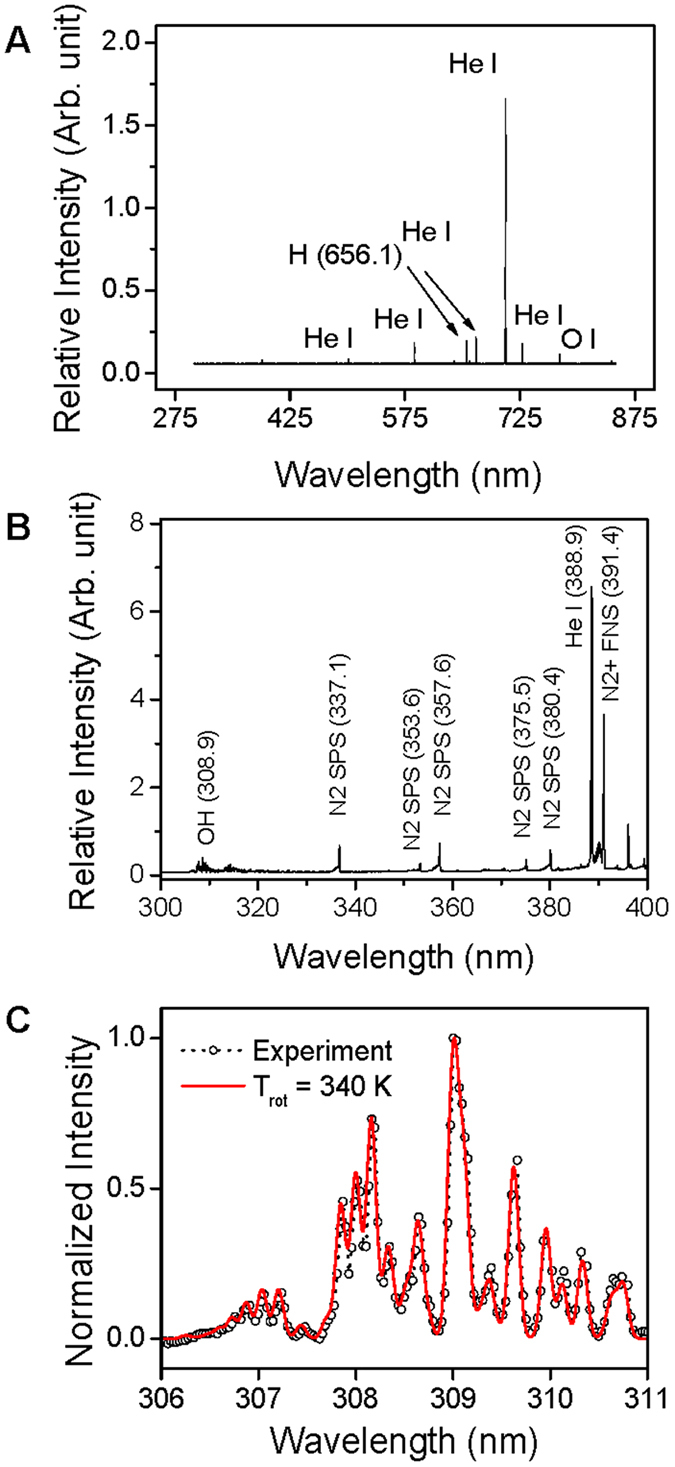
(**A**) Atomic emission spectral lines in a range between 300 and 850 nm. Helium (He I), oxygen (O I), and hydrogen (H I) atom emission lines are dominant. (**B**) Molecular emission spectral lines between 300 and 400 nm. N_2_ SPS, N_2_^+^ FNS, and OH molecular lines can be seen. (**C**) Rotational temperature is calculated as 340 K from the measured OH lines. (*See color version online.*).

We employed atmospheric-pressure dielectric barrier discharge plasma to investigate its anticancer effects on CRC cell lines HT29 and HCT116 (Fig. 1). Characteristics of the discharge were investigated by means of electrical measurements (Fig. 2) and optical emission spectroscopy. Maximal voltage was applied unless high-current streamers arose (Fig. 2B). This condition also allowed uniform plasma treatment over the entire electrode area, which is appropriate for treating CRC cells in a Petri dish (Fig. 3C). In the optical emission spectra, typical products of RONS, such as O I, OH, N_2_, and N_2_^+^ molecular lines, were clearly observed (Fig. 4A,B). A low gas temperature of 340 K was measured using OH molecular spectra analysis to ensure that the plasma treatment did not cause considerable thermal damage to the cells (Fig. 4C).

As noted above, plasma produces charged particles, reactive radicals, and light emissions in various ranges. Among these, charged particles such as electrons and ions obviously influence cell viability when their flux is sufficiently large. However, the effect of charged particles was expected to be insignificant, because we generated a type of surface discharge rather than a volume discharge in our system. Another possible consideration would be emission lines from the discharge, because UV lamps are frequently used to sterilize bacteria^41^. Even though we observed OH molecular emission lines near 309 nm, they were far from the wavelength of mercury UV lamps (253.7 nm). Moreover, significant UV lines shorter than 300 nm were not detected in our experiment. It is strongly believed that, eventually, reactive radical species influence cellular biological systems.

Previously, the anticancer effects of atmospheric-pressure plasma on human colon cancer cells have been studied by several research groups^42^,^43^,^44^. For example, cell lines for both colorectal carcinoma (HCT116) and adenocarcinoma (HT29, LoVo, DLD-1, and HCT15) were investigated^42^, focusing on the expression level of p53 and antiproliferative effects. Plasma inhibition of cell migration and invasion was observed for the colon cancer cell line SW480; on the other hand, these effects were not significantly observed in a condition of gas flow without the discharge^44^. The arrest of cell growth was effectively demonstrated in the HCT116 cell line by means of cell-cycle analysis (Western blotting of cell cycle–related genes), which suggested that pp38, pJNK, and pERK expressions were activated, as was phosphorylation of β-catenin, by plasma^44^.

β-catenin is an important transcription factor involved in cell adhesion; however, Sp1 plays an important role as a basal transcription factor and represents an essential promoter in cancer cells^45^. For this reason, numerous studies of Sp1 in colon cancer cells and a variety of other cancer cells have been reported in the chemotherapeutic literature^14^,^15^,^46^,^47^,^48^, although few studies have dealt with Sp1 in terms of the anticancer effects of atmospheric-pressure plasma. The level of Sp1 expression is higher in cancer cells than in normal cells^49^. Also, the downregulation of Sp1 has resulted in effective growth of cancer cells in nude mice^50^. Therefore, downregulating Sp1 is a good strategy for preventing tumor cell growth.

**Figure 5:**
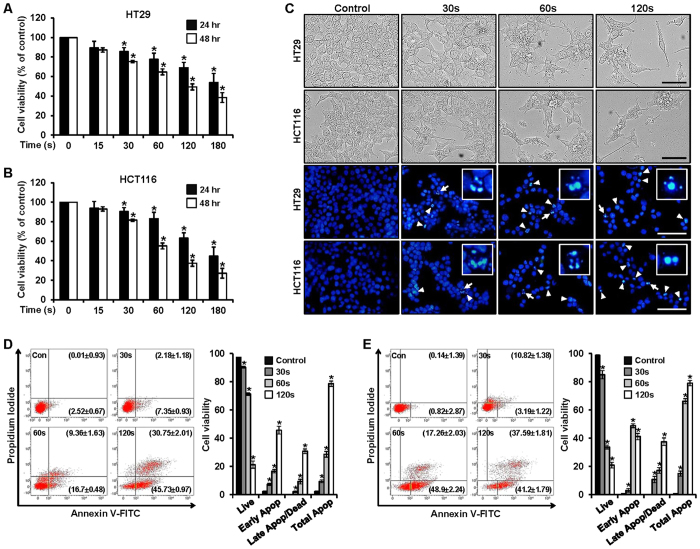
(**A,B**) Cell viability in plasma-treated HT29 and HCT116 cells (at 15, 30, 60, 120, and 180 sec) was detected using an MTS assay kit. Data represent mean percentage levels ± standard deviations (SD). *Significantly different, as compared with untreated controls, by the paired t-test (n = 3; p < 0.05). (**C**) Morphological changes observed in the plasma-treated HT29 and HCT116 cells (for 30, 60, and 120 sec) and the untreated cells. Fluorescence microscopic images of DAPI-stained cells. Left-hand image shows identification of DNA fragmentation by fluorescent DAPI assay (white arrows head). Right-hand image with white arrows that indicate DNA fragmentation and quantification of DNA fragmentation, scale bar = 200 μm. (**D**) HT29 and (**E**) HCT116: Quantitative detection of Annexin V-FITC and PI positive cells using means of fluorescence-activated cell sorting (FACS). HT29 and HCT116 cells were treated with plasma, and apoptosis was analyzed via Annexin V-FITC and PI staining. Data represent mean percentage levels ± SD (n = 3; *P  < 0.05). *(See color version online.).*

**Figure 6:**
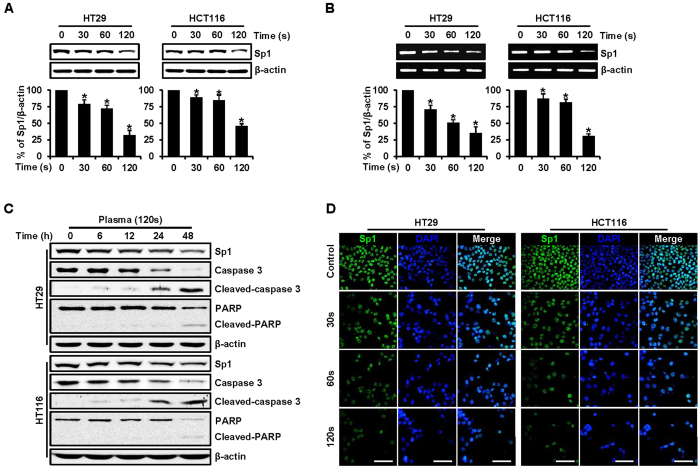
(**A**) HT29 and HCT116 cells were treated with plasma for 30, 60, and 120 sec, and whole-cell extracts were prepared, separated on SDS-PAGE, and subjected to Western blotting analysis against Sp1 antibody. Actin was employed as a loading control. The graphs indicate the ratio of Sp1–to-actin expression. (**B**) Effects of plasma (for 30, 60, and 120 sec) on Sp1 mRNA. The graphs indicate the ratio of Sp1-to-actin expression. Data represent mean percentage levels ± SD (n = 3; *p < 0.05). (**C**) Experiments to assess dose time–dependent effects of plasma on Sp1, caspase 3, cleaved caspase 3, PARP, and cleaved PARP were carried out using HT29 and HCT116 cells treated with plasma (120 sec) at 6, 12, 24, and 48 h. Actin was employed as a loading control. (**D**) Immunofluorescence microscopy of HT29 and HCT116 cells treated with plasma (for 30, 60, and 120 sec). Cells were immunostained with anti-Sp1, and signals were detected with 488-conjugated secondary antibody. DAPI was used for nuclear counterstaining. Data are expressed as means ± SD of three independent experiments, scale bar = 100 μm. (*See color version online.*).

In our study, the anticancer effects of PDBD on the CRC cell lines was mainly investigated in a dose time–dependent manner. Evolution of the expression levels of Sp1 and of regulatory proteins closely related to apoptosis and cell cycles was observed by comparing results in untreated (control) cells and plasma-treated cells. We used MTS assay, DAPI and PI staining, and annexin V–FITC staining to classify cell viability into live, early apoptosis, late apoptosis, and dead. In both the HT29 and HT116 cell lines, total apoptosis was increased, and the percentages of otherwise live cells were reduced with increasing dose times (Fig. 5). Abnormal DNA fragmentation was also observed on DAPI-staining (Fig. 5C). These apoptotic effects were studied by means of Western blotting and RT-PCR analyses, in which the expression levels of Sp1, caspase 3, PARP, cleaved caspase 3, and cleaved PARP coincided with the results of the cell viability tests and gave detailed insights into these effects (Fig. 6A,B). Based on our findings, the apoptotic effect was induced by certain PDBD dose times.

**Figure 7:**
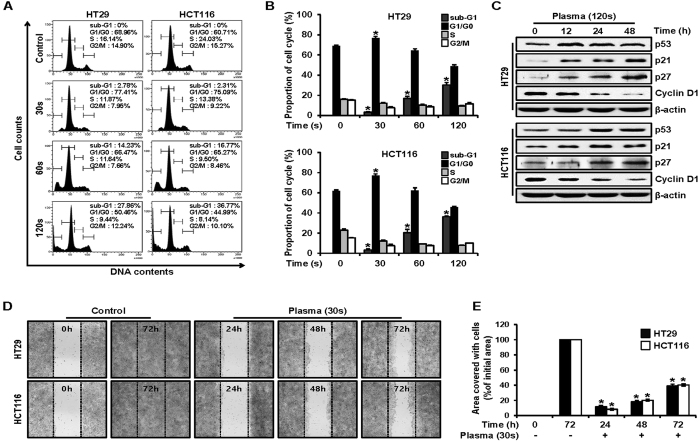
(**A**) HT29 and HCT116 cell cultures were treated with plasma (for 30, 60, and 120 sec) or untreated (control cells), and the cells were washed, fixed, stained with PI, and analyzed for DNA content by FACS analysis. The percentage of apoptotic cells was measured by FACS analysis after PI staining. (**B**) Flow cytometry was quantified. Data represent mean percentage levels ± SD (n = 3; *p < 0.05). (**C**) HT29 and HCT116 cells were treated with plasma (120 sec) and examined at 12, 24, and 48 h, and whole-cell extracts were prepared, separated on SDS-PAGE, and subjected to Western blotting using p53, p21, p27, and cyclin D1 antibodies. Actin was employed as a loading control. The results represent three independent experiments. (**D**) HT29 and HCT116 cells were treated with plasma (for 30 sec), and confluent cells were carefully scratched using sterile pipette tips and then re-cultured with or without plasma. At 24, 48, and 72 h, the cells were photographed under a microscope. (**E**) Migration assay was quantified. Data represent mean percentage levels ± SD (n = 3; *p < 0.05).

**Figure 8:**
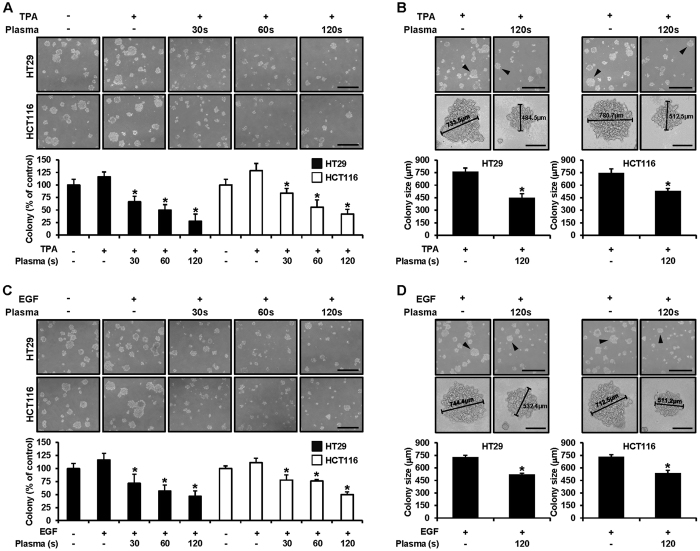
(**A**,**B**) HT29 and HCT116 cells were treated with tetradecanoyl phorbol acetate (TPA) and plasma (for 30, 60, and 120 sec) in 1 mL of 0.3% basal Eagle’s medium containing 10% FBS for the anchorage-independent cell transformation assay. Both types of cells were incubated at 37 °C in a 5% CO_2_ incubator for 14 days, and the number of colonies was counted. Colony counts and sizes are expressed as means ± SD (n = 3; *p < 0.05 vs. control cells), scale bar = 400 μm. (**C**,**D**) HT29 and HCT116 cells were treated with epidermal growth factor (EGF) and plasma (for 30, 60, and 120 sec) in 1 mL of 0.3% basal Eagle’s medium containing 10% FBS for the anchorage-independent cell transformation assay. Both types of cells were incubated at 37 °C in a 5% CO_2_ incubator for 14 days, and the number of colonies was counted. Colony counts and sizes are expressed as means ± SD (n = 3; *p < 0.05 vs. control cells).

To better understand these results, we examined Sp1 target proteins, including p53, p21, p27, and cyclin D1^51^. Cell-cycle arrest was estimated based on the measurement of DNA content with regard to a specific phase of the cell cycle and showed an increase in sub-G1 with an increase in dose time (Fig. 7A,B). The kinases of p53, p21, and p27 are negative regulators of the cell cycle^52^,^53^, and these were upregulated, indicating that cell-cycle arrest blocked progress to the next step in the cycle (Fig. 7C). On the other hand, cyclin D1 is a positive regulator involved in oncogenesis^54^. Thus, both positive and negative regulators definitely indicated cell-cycle arrest, thus increasing the sub-G1 phase. Lastly, colony formation was observed with or without the presence of tumor promoters such as TPA and EGF, which resulted in the suppression of colony formation by plasma in terms of both size and quantity (Fig. 8).

In conclusion, we demonstrated that atmospheric-pressure dielectric barrier plasma induced apoptosis and cell-cycle arrest, accompanied by the downregulation of Sp1 expression, in two human colorectal cancer cell lines, HT29 and HCT116. This study supports the possibility of regulating transcription factor Sp1 in cancer cells by treatment with atmospheric-pressure plasma, which could represent a potential application in anticancer therapy.

## Materials and Methods

### Atmospheric-pressure plasma source and diagnostic system

Figure 1 depicts the experiment in schematic form. A standard size Petri dish (100 mm in diameter) was successfully combined with the PDBD source. A disc-shaped copper electrode was encapsulated inside the dielectric material, which was electrostatically coupled to the source body of stainless-steel through a dielectric window of quartz. Since the PDBD is a kind of surface discharge owing to its geometrical electrode shape, the plasma is only produced on the surface of top electrode area. Four holes which were symmetrically placed in the edge of the reactor were made on the top of the source: two for the gas feed and the other two for exhaust. A 50-kHz power was applied to the PDBD, with a duty ratio of 50% and a pulse width of 5 μsec. For the plasma–cell interaction, 1 kV of V_rms_ was applied because the glow discharge uniformly appeared without high-current streamers in this condition. Both the voltage applied to the electrode and the current passing through a serial circuit were measured by means of a high-voltage probe (PPE 20KV Teledyne, LeCroy, Santa Clara, CA, USA) and a current probe (wideband current monitor 6585, Pearson Electronics, Palo Alto, CA, USA), which were monitored through a digital oscilloscope (44MXs-B, Teledyne LeCroy). To analyze optical emission spectra from the discharge, optical fiber was placed beside the discharge and combined with a spectroscope (IsoPlane SCT 320, Princeton Instruments, Acton, MA, USA) equipped with a charge-coupled device (PIXIS 400 CCD camera, Princeton Instruments).

### Cell lines and culture conditions

The colorectal cancer cell lines HT29 (ATCC HTB-38) and HCT116 (ATCC CCL-247) were obtained from the American Type Culture Collection (ATCC, Manassas, VA, USA). HT29 was derived from tumor tissue obtained from a patient prior to the initiation of chemotherapy, and HCT116 was established from lymph node metastases obtained from the lung of a patient who had undergone radiation therapy. These colorectal cancer cell lines were grown routinely in DMEM medium (Welgene, Deagu, Korea) with 10% fetal bovine serum (FBS) and 100 U/mL each of penicillin and streptomycin (Gibco, Grand Island, NY, USA) at 37 °C with CO_2_ in a humidified atmosphere.

### MTS cell viability assay

The effects of plasma on cell viability were estimated using an MTS Assay Kit (Promega, Madison, WI, USA). After the plasma treatment, MTS solution was added to each well for 2 h at 37 °C in 5% CO_2_. The absorbance at 490 nm was recorded using a GloMax-Multi+ Microplate Multimode Reader (Promega).

### DAPI staining

The levels of nuclear condensation and fragmentation were observed by means of nucleic acid staining with DAPI. The HT29 and HCT116 cells treated with plasma were harvested by trypsinization and fixed in 100% methanol at room temperature for 20 min. The cells were seeded on slides, stained with DAPI (2 μg/mL), and monitored by means of confocal laser microscopy (FluoView FV10, Olympus, Tokyo, Japan).

### Annexin V–FITC assay and propidium iodide staining

Apoptosis can be evaluated by means of simultaneous staining with annexin V–FITC and propidium iodide (PI). Annexin V–FITC staining reveals the early stage of apoptosis, and PI staining shows the late stage. The HT29 and HCT116 cells were incubated for various plasma dose times (30, 60, and 120 sec) and then incubated for 48 h, after which the cells were harvested using a scraper. The harvested cells were stained with annexin V–FITC and PI and then assessed by means of fluorescence-activated cell sorting (FACS, BD Biosciences, San Jose, CA, USA).

### Immunocytochemical testing

The HT29 and HCT116 cells were seeded over each sterilized glass coverslip on six-well tissue culture plates for 24 h, treated with plasma (for 30, 60, and 120 sec), and then incubated for 48 h. The cells were then fixed and permeabilized with Cytofix/Cytoperm solution (BD Biosciences, San Jose, CA, USA) for 30 min. For Sp1 expression, the cells were blocked with 0.5% bovine serum albumin and then incubated with a monoclonal Sp1 antibody at 4 °C overnight. After the cells were washed with PBST (PBS containing 0.1% Tween-20) solution, the Sp1 antibodies were reacted with the Alex Fluor 488–conjugated anti-mouse secondary antibody (Jackson ImmunoResearch, West Grove, PA, USA) at room temperature for 1 h and then mounted onto the cells with the Vectashield mounting medium for fluorescence with DAPI (Vector Laboratories, Burlingame, CA, USA). These cells were visualized using the FluoView confocal laser microscope (Olympus, Tokyo, Japan).

### Western blot analysis

The HT29 and HCT116 cells were treated with plasma (for 30, 60, and 120 sec) and then incubated for 48 h, washed with PBS, and then lysed with M-PER Mammalian Protein Extraction Reagent (Thermo Scientific, Rockford, IL, USA). Extracted proteins were quantified using the Pierce BCA Protein Assay Kit (Thermo Scientific). Equal amounts of the protein samples were separated by 10% or 15% SDS–polyacrylamide gel electrophoresis and then transferred to membranes. The membranes were blocked for 1 h at room temperature with 5% non-fat dried milk in PBS containing 0.1% Tween-20 and then incubated overnight at 4 °C with specific antibodies. Protein bands were observed after treating the membranes with horseradish peroxidase–conjugated secondary antibody using a Pierce ECL Western Blotting Substrate (Thermo Scientific).

### Cell-cycle analysis

The HT29 and HCT116 cells treated with plasma (for 30, 60, and 120 sec) were incubated for 48 h and washed with cold PBS, pooled, and centrifuged before being fixed in 70% ice-cold ethanol overnight at −20 °C. They were then treated with 100 μg/mL of RNase A and 40 μg/mL of propidium iodide. The stained cells were analyzed, and their distribution in different phases of the cell cycle was calculated using flow cytometry by means of FACS (BD Biosciences).

### Wound healing assay

A scratch wound healing assay was performed as previously described^55^,^56^. Briefly, the HT29 and HCT116 cells were grown to confluence on 100-mm-diameter culture dishes. The monolayer was scratched with a sterile pipette tip and was then washed with PBS to remove cellular debris. Cells were then grown in DMEM containing 10% FBS with plasma (for 30 sec). At 24, 48, and 72 h, cell migration was observed under a microscope and photographed.

### Anchorage-independent cell transformation assay

Both the HT29 and HCT116 cells (1.2 × 10^4^ per well) were suspended in 1 mL of BME, 10% FBS, and 0.3% agar and plated with plasma for various lengths of time (30, 60, and 120 sec). They were then incubated for 48 h on 3 mL of solidified BME containing 10% FBS and 0.5% agar for 14 days. Colony numbers and sizes were measured under a microscope.

### Statistical analysis

The results are presented as means  ± SD for at least three independent experiments performed in triplicate. Data were analyzed using one-way analysis of variance, with p < 0.05 indicating statistical significance.

## Additional Information

**How to cite this article**: Han, D. *et al*. Antitumorigenic effect of atmospheric-pressure dielectric barrier discharge on human colorectal cancer cells via regulation of Sp1 transcription factor. *Sci. Rep.* **7**, 43081; doi: 10.1038/srep43081 (2017).

**Publisher's note:** Springer Nature remains neutral with regard to jurisdictional claims in published maps and institutional affiliations.
